# Urban–rural disparity in the relationship between ambient air pollution and preterm birth

**DOI:** 10.1186/s12942-020-00218-0

**Published:** 2020-06-20

**Authors:** Long Li, Jing Ma, Yang Cheng, Ling Feng, Shaoshuai Wang, Xiao Yun, Shu Tao

**Affiliations:** 1 Beijing Key Laboratory for Remote Sensing of Environment and Digital Cities, Faculty of Geographical Science, Beijing Normal University, Beijing, 100875 China; 2 Tongji Hospital of Tongji Medical College, Huazhong University of Science and Technology, Wuhan, 430030 China; 3 College of Urban and Environmental Sciences, Peking University, Beijing, 100871 China

**Keywords:** Air pollution exposure, Preterm birth, Urban–rural disparity, Spatial correlation, Multilevel logistic model

## Abstract

**Background:**

Some studies have reported that air pollution exposure can have adverse effects on pregnancy outcomes. However, the disparity between urban and rural areas in the risk of preterm birth (PTB) has yet to be elucidated. Considering geographic contexts as homogeneous or ignoring urban–rural differences cannot accurately reveal the disparities in the health effects of air pollution under different geographic contexts. The aims of this study were to examine the disparities in the risks of PTB in three different regions and five urban–rural types and to investigate the extent to which fine particulate matter (PM_2.5_) exposure during the entire pregnancy can explain the variations.

**Methods:**

We collected data on 429,865 singleton newborns born in 2014 in Hubei Province, China, and divided Hubei Province into three regions. Spatial correlation methods were employed to measure the associations between the rate of PTB and air pollution using average annual indexes for the entire province and regions. A series of multilevel logistic models were conducted to examine disparities in the risks of PTB with decreases in urbanity and the effects of air pollution exposure on the occurrence of preterm births.

**Results:**

The PM_2.5_ concentration was significantly different across the regions. The eastern region had the most wide-ranged and serious level of pollution, whereas the levels in the middle and western regions weakened. The odds of PTB and air pollution exhibited a positive spatial correlation for the entire province and in the east (*BiMoran’s I* = 0.106 and 0.697, respectively). Significant urban–rural disparities in the risks of PTB were noted in the east and middle regions, and the mean PM_2.5_ exposure during the entire pregnancy was positively associated with PTB risk. However, in the west, the results showed weak differences in the risks of PTB among the five urban–rural types and an insignificant effect of PM_2.5_ exposure. The direction of the effect of district/county-level income on PTB varied by region.

**Conclusions:**

This study finds that air pollution exposure and PTB have significant and positive spatial relationships in areas with a serious air pollution burden. The risks of PTB in three regions of Hubei Province follow the same W-shaped pattern as urbanity decreases and rurality increases. High levels of air pollution exposure may be an important disadvantage for urban pregnant women in this setting.

## Background

Fine particulate matter (with an aerodynamic diameter ≤ 2.5 μm, PM_2.5_) has become a main pollutant, and its relationship with human health has attracted great attention worldwide [1, 2]. With potential biological mechanisms related to inflammation and oxidative stress [3–5], PM_2.5_ can induce cardiovascular disease and respiratory disease [6–10], and increase the mortality rate [11, 12]. Moreover, PM_2.5_ can prevent people from engaging in outdoor physical activities [13, 14] and has adverse effect on mental health, such as depression and anxiety [15–17].

The relationship between air pollution and delivery outcomes has become an important topic. Gestational week and birth weight are important predictors of infant morbidity and mortality [18–20]. Considerable research has explored the relationships between maternal air pollution exposure and various adverse outcomes, such as preterm birth (PTB), low birth weight (LBW), and small for gestational age (SGA). However, the results are inconclusive. While some studies showed that long-term or short-term maternal exposure during pregnancy had positive influences on adverse delivery outcomes [5, 21–25], other studies found insignificant relationships [26] or even negative associations [27].

In addition to differences in methods [28, 29], the inconsistent results are also related to spatial landscape heterogeneity. Past cross-country or regional studies revealed spatial variations in exposure-health relationships [30, 31]. Landscape heterogeneity refers to the differences in certain characteristics of study areas, such as lifestyles, socioeconomic status (SES) and ecological environment, and these characteristics could mediate the relationship between pollution exposure and birth outcomes. Some research has suggested the notion of “double jeopardy”, wherein the effect of the environment on health is greater in poor areas [32, 33]. To make matters more complicated, the economy-emission relationship based on the environmental Kuznets hypothesis potentially exhibits an inverted U-shaped pattern [34]; thus, the impacts of income increases on health are not always positive.

The introduction of urban–rural location types could account for different economy-environment contexts. Compared with their urban counterparts, rural residents generally have disadvantaged SES, low nutritional status and less convenient access to medical services [35–38]. Some research showed that urban residents have better physical health than rural people [39, 40], while other research found non-significant distinctions [41]. Urban–rural disparities in environment-health relationships have been examined in very few studies [42, 43]. For instance, a recent study found that rural mortality was more sensitive to adverse temperature conditions than urban mortality [42]. Moreover, some research further indicated that health status did not change monotonically along the urban–rural continuum but more likely followed an inverted U or J-shape [44–46]. In terms of birth outcomes, Larson et al. found that crude rates of LBW in non-metro areas were lower than those in metropolitan areas in the US [47], while some other studies reported that pregnant women in rural areas adjacent to urban areas (or suburban areas) had better birth outcomes than both remote rural and urban counterparts [48, 49]. By and large, the urban–rural disparity in the relationship between air pollution exposure and PTB has yet to be elucidated. In most prior studies, the urban–rural dichotomy assumed that one was superior to the other, ignoring the heterogeneity among metropolis, small cities, large towns, and rural areas, which could greatly differ in air quality, SES and health. Thus, a finer classification of urban–rural areas should be made in research on the relationships between PTB and environmental pollution.

This study attempts to investigate the air pollution-PTB association with a finer urban–rural classification using the case of Hubei Province, China. First, we investigated whether exposure to PM_2.5_ during the entire pregnancy significantly increased the risk of PTB. Second, we examined the disparities in the risks of PTB among various urban–rural continua and how air pollution exposure explained these variations. To the best of our knowledge, this is the first study that examines whether and to what extent PM_2.5_ exposure can explain the PTB risks with a finer-scale analysis of both urban–rural and regional disparities. This study contributes to the environmental health literature worldwide and provides a better understanding of the urban–rural disparity in the relationships between ambient air pollution exposure and PTB, particularly in developing countries, where such research has been very scarce to date.

## Study area

**Fig. 1:**
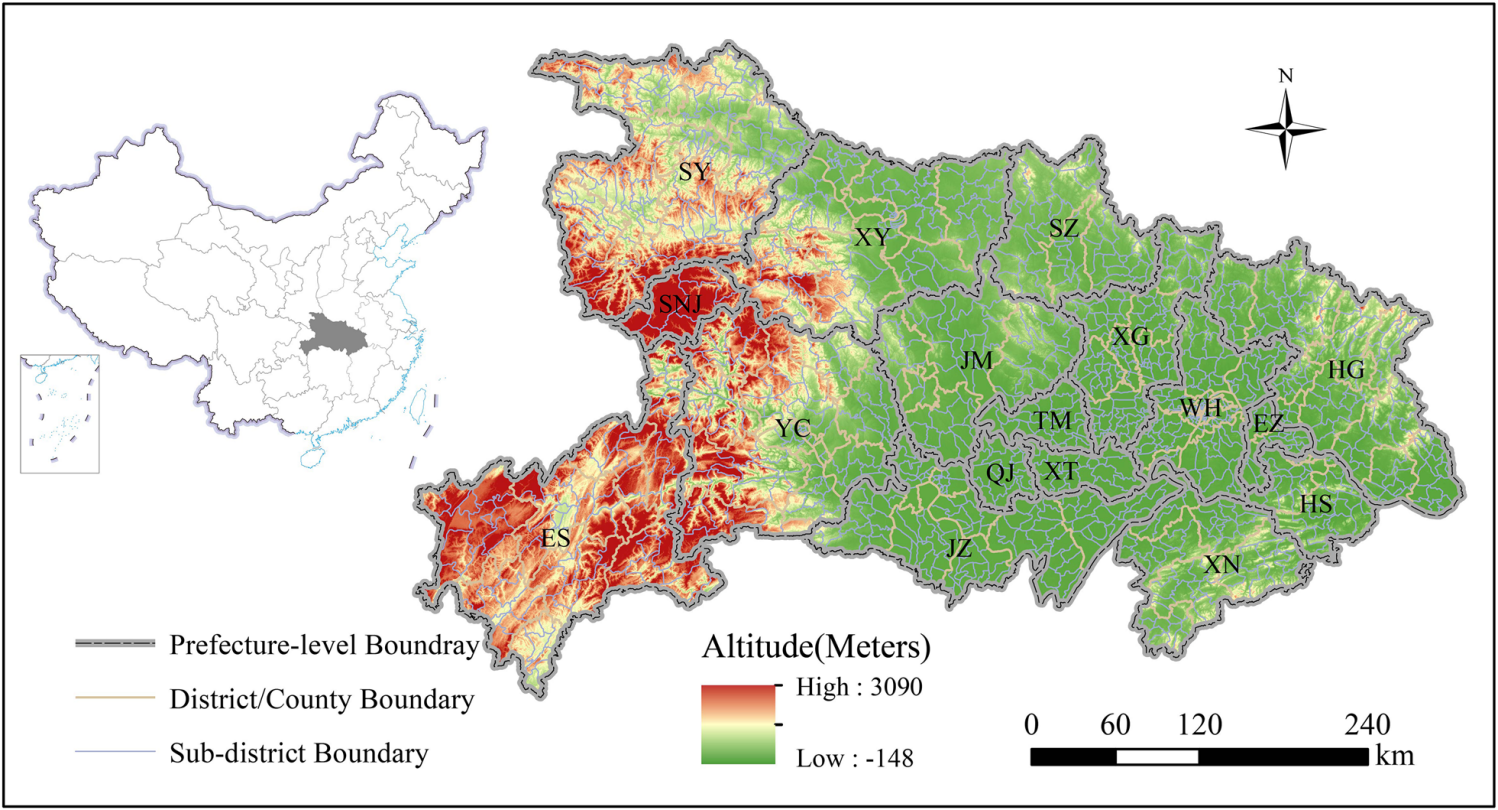
Location of Hubei Province and its terrain and administrative division

Figure 1 shows the location of Hubei Province, which includes 17 prefecture-level cities, and each city consists of one or more districts and counties. The total area of the province is approximately 185,900 km^2^, and the population was 58.16 million by the end of 2014. Regarding natural conditions, mountainous regions account for 56% of the province [50] and are especially concentrated in the west, which partly restricts the development of urbanization in these areas and greatly affects the population and economic distribution of the province.

## Data

### Neonatal and maternal clinical data

In the analysis, the birth records of newborns and information on pregnant women were collected from the Health Commission of Hubei Province, and data from a total of 557,243 newborns in 2014 were obtained. The dataset records the maternal residence, hospital of delivery, individual and household socio-economic information, physical examinations during pregnancy, neonatal and maternal physiological characteristics at the time of delivery, and so on. We geocoded each pregnant woman’s residence location at the sub-district level, and 485,306 newborns with complete maternal residence information were retained. For each newborn, we collected considerable information on his/her mother, such as age, race/nationality, educational level, pregnancy test, conception and labour date. Neonatal characteristics, including the birth date, gender, birth weight and gestational age (GA), were collected. The GA was calculated by the delivery date minus the last menstrual period (LMP). Newborns with missing information and non-singletons were removed. According to clinical definition, less than 37 completed gestational weeks is considered a preterm birth. Moreover, we also excluded samples with GA less than 32 weeks or greater than 44 weeks and birth weight less than 1.0 kg or more than 5.5 kg. Finally, we selected 429,865 singleton newborns for analysis.

### Air pollution data

We employed the *Weather Research and Forecasting Model Coupled with Chemistry* (WRF/Chem, version 3.5) [51] to simulate the daily PM_2.5_ concentration in Hubei Province and its surrounding areas. The initial meteorological data in 2014 were extracted from the National Centres for Environmental Prediction Final Operational Global Analysis data (http://rda.ucar.edu/datasets/ds083.2/). Simulated meteorological data generated by WRF modelling were validated by meteorological data from China Earth International Exchange stations. The modelled PM_2.5_ concentrations were then downscaled to a resolution of 1 km × 1 km using a Gaussian downscaling method based on a PM_2.5_ emissions inventory and wind data (http://inventory.pku.edu.cn/) and validated by air quality monitoring (AQM) data (http://beijingair.sinaapp.com/). For further details on the model evaluation, please see Shen et al. [52].

The daily PM_2.5_ concentrations in 2014 at the sub-district level were calculated by overlaying the sub-district administrative shapefile of Hubei and 1 km × 1 km grid data using ArcGIS 10.3. The formula is shown in Eq. (1):

1 $$C_{k} = \mathop \sum \limits_{i = 1}^{m} \frac{{S_{ik} }}{{S_{k} }}C_{i}$$

where *C*_*k*_ and *C*_*i*_ represent the daily average PM_2.5_ concentrations in sub-district *k* and grid *i*, respectively; *m* is the total number of grids with intersections with sub-district *k*; *S*_*ik*_ is the area of the corresponding intersection; and *S*_*k*_ is the total area of sub-district *k*.

**Fig. 2:**
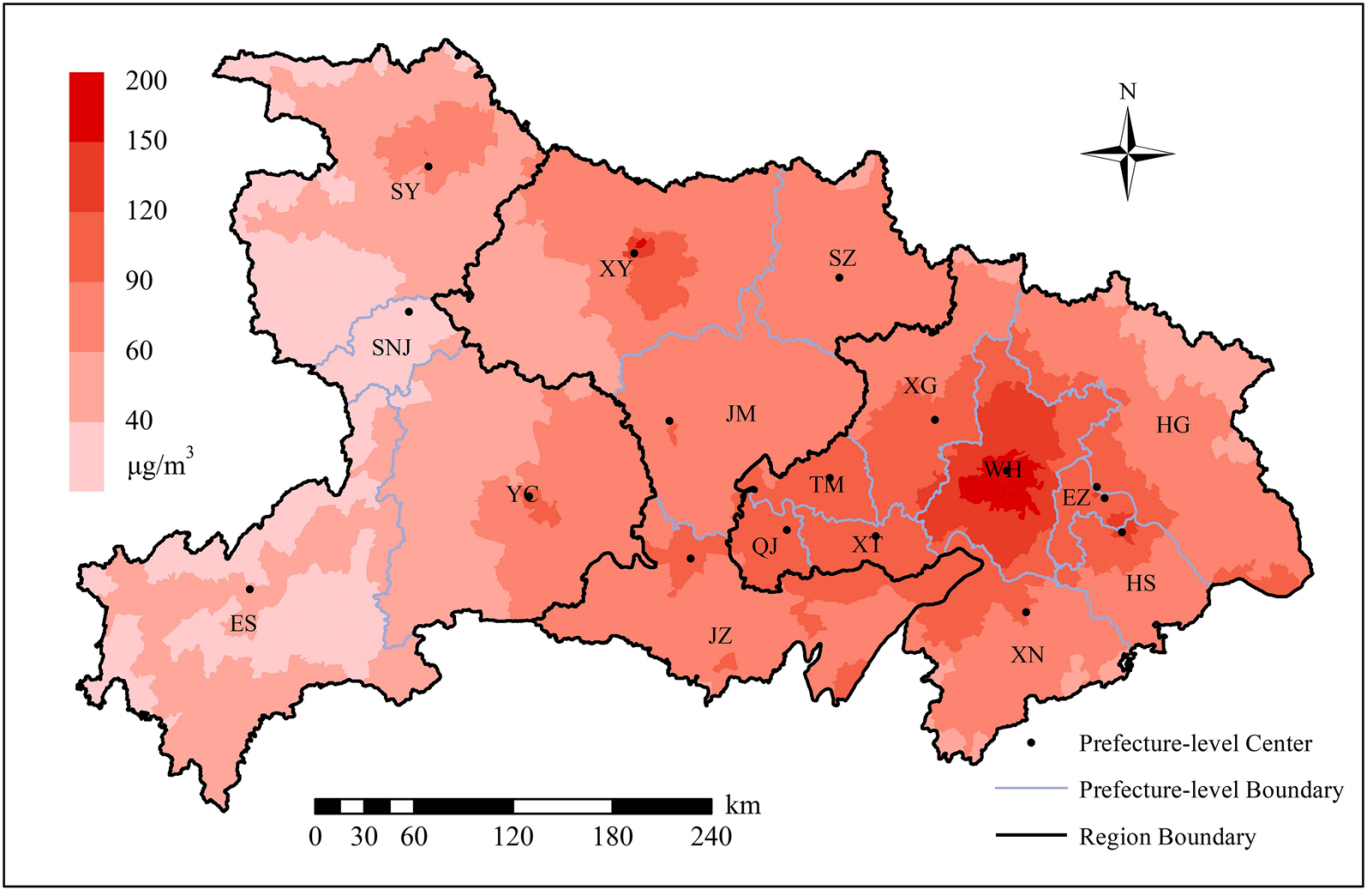
Spatial distribution of annual PM_2.5_ concentration at the sub-district level in 2014 in Hubei Province

Given that AQM data before 2014 are not available in Hubei Province, we only simulated the daily PM_2.5_ concentrations in 2014 and replaced 2013 concentrations using the same period for each sub-district. Given that a pregnancy term covers approximately 75% of a year and considering the seasonality of air pollutants [53], calculating the pollution exposure during the entire pregnancy is superior to using an annual average concentration [2, 33]. After the daily concentrations were averaged, the spatial distribution of the annual PM_2.5_ concentrations in 2014 at the sub-district level in Hubei Province was determined, as shown in Fig. 2.

### Area-level income data

Family income was not available in the birth records, and thus we instead collected annual income at the district/county level from the *Hubei Statistical Yearbook* 2014 [54]. The income data of each district/county in the yearbook consisted of two columns, urban per capita disposable income (UDI) and rural per capita net income (RNI), which represent the income status of urban and rural residents in this district/county, respectively. We assigned UDI to pregnant women living in a city proper or county town and RNI to three other urban–rural types (See “Methods”).

## Methods

### Regional division and definition of urban–rural continuum

**Fig. 3:**
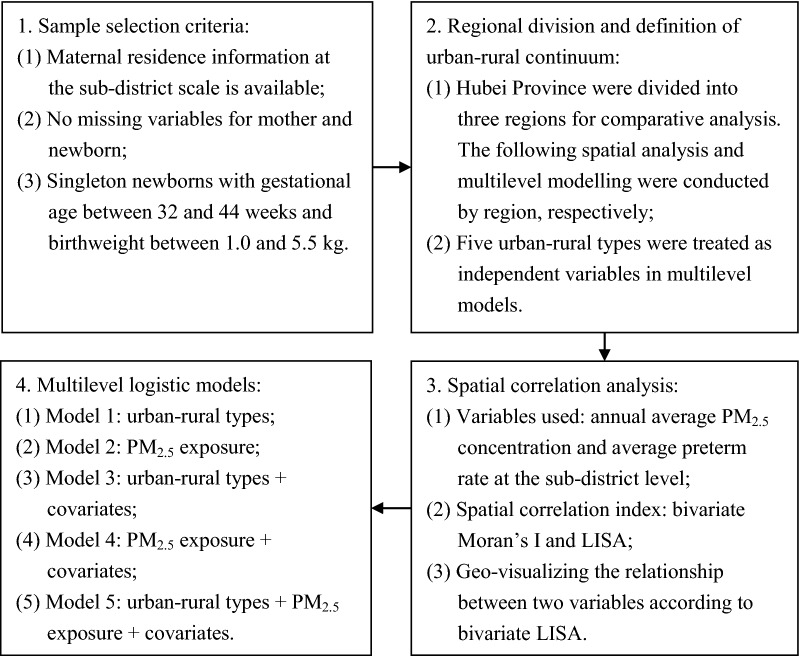
Overview of the methodology in this study

Figure 3 provides an overview of the methods used in this study. First, we divided Hubei Province into three regions for comparative analysis. The east includes Wuhan (WH), Xianning (XN), Huangshi (HS), Huanggang (HG), Ezhou (EZ), Xiantao (XT), Xiaogan (XG), Tianmen (TM) and Qianjiang (QJ). The middle includes Jingzhou (JZ), Jingmen (JM), Xiangyang (XY) and Suizhou (SZ). The west includes Enshi (ES), Yichang (YC), Shiyan (SY) and Shennongjia (SNJ). Heterogeneity of physical and socioeconomic conditions was noted among these three regions. The east is the focus of economic development in the province and is also called Wuhan Metropolitan Area, while the terrain in the west is mainly mountainous with a sparse population and under-developed economy (Fig. 1).

We also defined five types of urban–rural continua according to the Urban–Rural Classification and Codes (URCC) by the China National Bureau of Statistics using the indicators of population and economic development [55]. The URCC includes three main urban–rural categories: district (code: 110), township (120) and countryside (200). Sub-district is the smallest administration cell and census tract in China and include several types, such as *jiedao*, *zhen*, and *xiang*. All sub-districts in the districts are called *jiedao* (city proper, i.e., the most urbanized areas of central cities) or *zhen* (suburb, i.e., areas adjacent to city proper). Township refers to the *zhen* in the counties and can be further divided into two types: county town (121) and general town (122). The county town is the seat of the county government, and the urbanized areas in this location are inferior to the city proper but superior to the suburbs. In addition, counties also include other areas called *xiang*, which is the countryside. Thus, we finally divided all sub-districts into five types of urban–rural continua: city proper, county town, suburb, general town and countryside (Table 1), and this order reflects a decrease in urbanization and SES. Briefly, city proper and county town represent core urban areas with different scales, while suburbs, general town and countryside are different types of rural areas. Compared with suburbs, general town and countryside are more remote from urban areas, and the latter is a more deprived area than the former.

**Table 1:** The definition of urban–rural continuum

Code	Category	*jiedao*	*zhen*	*xiang*
*110*	*District*	City proper	Suburb	
*120*	*Township*			
121	County town		County town	
122	General town		General town	
*200*	*Countryside*			Countryside

### Spatial correlation analysis

Before regression modelling, we used a series of spatial correlation methods to measure and geo-visualize the spatial dependence between air pollution and the risk of PTB. The global spatial autocorrelation index is similar to Pearson’s correlation coefficient. However, the spatial adjacency is considered, and its general formula is measured by the Moran index (*Moran’s I*) in Eq. (2) [56, 57]:

2 $$Moran^{'} s I = \frac{{\mathop \sum \nolimits_{i = 1}^{n} \mathop \sum \nolimits_{j = 1}^{n} w_{ij} (x_{i} - \bar{x})\left( {x_{j} - \bar{x}} \right)}}{{S^{2} \left( x \right)\mathop \sum \nolimits_{i = 1}^{n} \mathop \sum \nolimits_{j = 1}^{n} w_{ij} }}$$

where *x*_*i*_ represents the attribute value of spatial unit *i*; $\bar{x}$ and *S*^*2*^ are the corresponding average and variance value of the entire region, respectively; *w*_*ij*_ is the element of the spatial weight matrix based on the first-order Rook contiguity method; and *n* is the total number of spatial units in the region.

The above index is applicable to single attribute. Bivariate *Moran’s I* (*BiMoran’s I*) measures the spatial correlation of two attributes by replacing *x*_*j*_ with *y*_*j*_, and the bivariate local indicator of spatial association (*BiLISA*) is used to measure this correlation between a spatial unit *i* and its neighbourhoods as noted in the following Eqs. (3) [58] and (4) [59]:

3 $$BiMoran^{'} s I = \frac{{\mathop \sum \nolimits_{i = 1}^{n} \mathop \sum \nolimits_{j = 1}^{n} w_{ij} (x_{i} - \bar{x})\left( {y_{j} - \bar{y}} \right)}}{{S\left( x \right)S\left( y \right)\mathop \sum \nolimits_{i = 1}^{n} \mathop \sum \nolimits_{j = 1}^{n} w_{ij} }}$$

4 $$BiLISA_{i} = z_{xi} \mathop \sum \limits_{j = 1}^{n} w_{ij} z_{yj}$$

where *z*_*xi*_ and *z*_*yi*_ represent standardized values (scaled by standard deviation) of attribute variables *x* and *y* of each spatial unit *i*; *S(x)* and *S(y)* are their standard deviation; and the other symbols are the same as noted in Eq. (2).

**Fig. 4:**
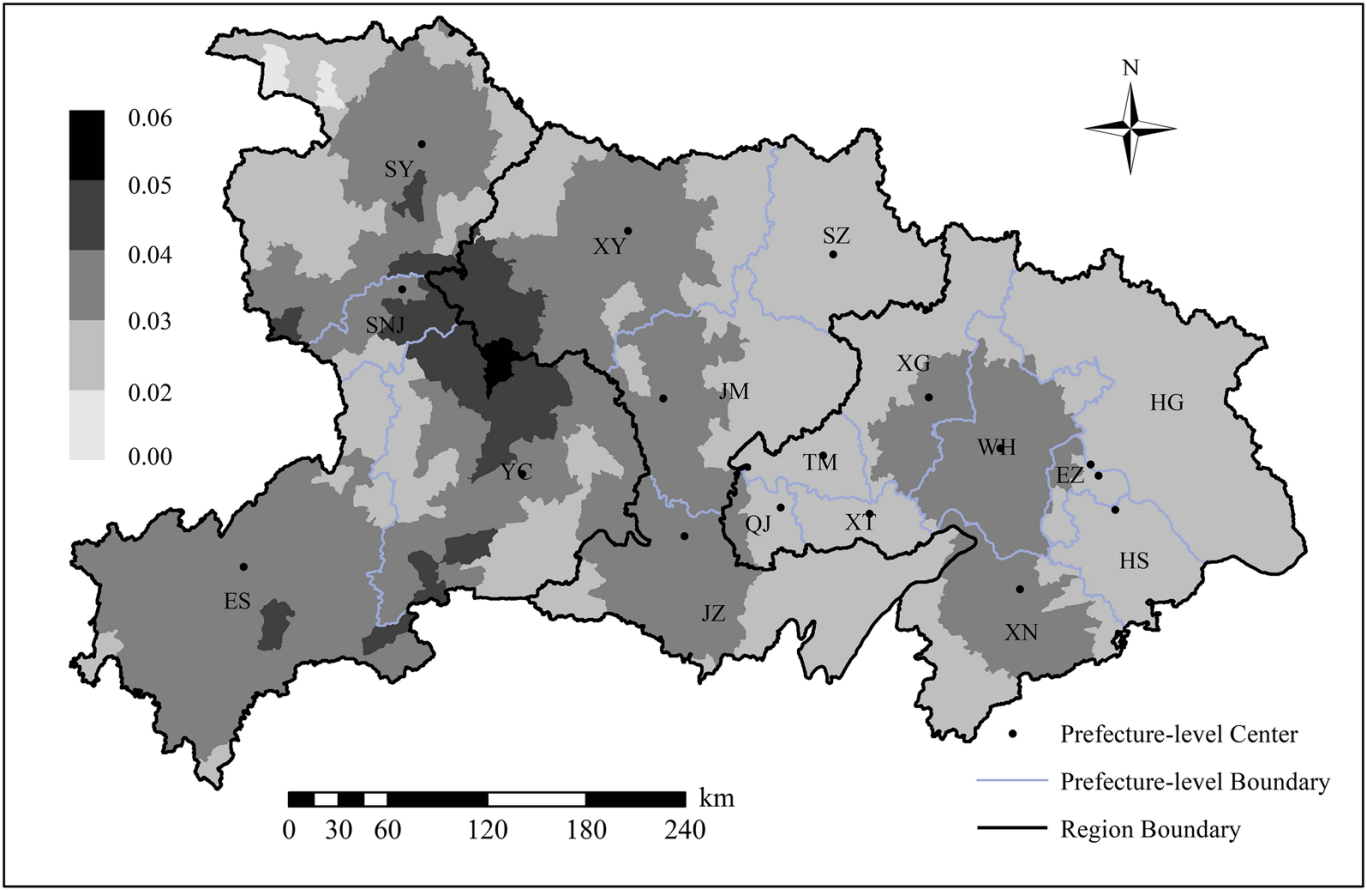
Distribution of preterm birth rate at sub-district level based on the spatial smoothing method in Hubei Province

Here, spatial units refer to each sub-district, and we used annual PM_2.5_ concentration as the explanatory variable *x*. Given the limited number of samples in many sub-districts, i.e., the small population problem [60], we constructed a smoothing window [61] to calculate the spatial average PTB rate as the dependent variable *y* for each sub-district. Specifically, we replaced sub-district polygons with their geometric centroids and took 50 km as the spatial window radius to calculate the average rate for each centroid. Figure 4 shows the spatial distribution pattern of the PTB rate in Hubei Province.

According to the *z*_*x*_, *BiLISA* value and its significance, the sub-districts can be divided into five categories: when *BiLiSA *> 0 and *p *≤ 0.05, a significantly positive spatial correlation is noted between air pollution and preterm rate, including types of H–H (when *z*_*x*_ > 0, i.e., high pollution with high preterm rate) and L–L (when *z*_*x*_ < 0, i.e., low pollution with low preterm rate); when *BiLiSA *< 0 and *p *≤ 0.05, a significantly negative correlation is noted, including types of H–L (when *z*_*x*_ > 0, i.e., high pollution with low preterm rate) and L–H (when *z*_*x*_ < 0, i.e., low pollution with high preterm rate); finally, when *p *> 0.05, there is no significant correlation [62]. We used Geoda [63] to calculate the above indexes for the whole province and three regions.

### Multilevel logistic model

Then, we used multilevel logistic models to analyse whether and to what extent air pollution exposure increased the risk of PTB during the entire pregnancy. Compared with a fixed logistic model, a multilevel model has a random effect to stratify samples to describe the potential group differences and unobserved variables. The general form of the model is as follows:

5 $$ln\left[ {\frac{{P\left( {y_{ij} = 1} \right)}}{{1 - P\left( {y_{ij} = 1} \right)}}} \right] = \beta_{0} + \mathop \sum \limits_{m = 1}^{p} \beta_{m} x_{ijm} + \mathop \sum \limits_{n = 1}^{q} \beta_{n} x_{jn} + u_{j} + \varepsilon_{ij}$$

where *P* is the probability of the event (for a premature infant, *y *= 1); *p* and *q* are the total number of variables in the level 1 and 2, respectively; and *u*_*j*_ is the random effect.

In this study, the random effect was based on the sub-district identifier. Explanatory variables included maternal residence types in the urban–rural continua, PM_2.5_ exposure and other individual-level and area-level covariates. PM_2.5_ exposure was measured as the average daily concentration of maternal permanent residence at the sub-district level during the whole pregnancy for each sample, which can prevent discussion of seasonal and long-term trends as we only used one year’s neonatal samples. The effects were reported by a 30-μg/m^3^ increase. Individual-level covariates contained categorical variables of maternal nationality (*Han* or minority), age (< 20, 20–24, 25–29, 30–34, or ≥ 35 years), educational level (primary, secondary, or tertiary), regular health or physical checks (less than 5 times or not), month of conception (January as reference), new-born sex (male or female), and parity (firstborn or non-firstborn). The area-level covariate referred to annual income at the district/county level and was scaled by provincial standard deviation. To test the possible non-monotonic relationship, we also added the squared term of income into models.

In the framework of multilevel models, we constructed a series of models. Model 1 contains only residence type variables to test the overall difference of PTB risk, while model 2 includes only the PM_2.5_ exposure variable. As controlled factors, individual- and area-level variables were added in models 3 and 4, respectively. Model 5 is the final model and included all variables mentioned above. Furthermore, to investigate whether the effects are varied, we also assessed all models using samples for each region. All of the multilevel models were tested using the lem4 package of the R software environment [64].

## Results

### Descriptive characteristics

Table 2 lists the samples of newborns used in this study and basic indicators of population and economy in 2014 for the entire province and each prefecture-level city. The capita GDP (GDP/Pop in Table 2) varies greatly in different cities. The values for WH, HS, EZ and QJ in the east, XY in the middle and YC in the west are greater than the provincial average, while the capita GDP in ES in the west is the lowest. The ratio of samples to total population is 7.39‰ for the whole province, which is close to the birth rate of Hubei Province in 2014 (11.86‰). Overall, our sample data are representative of newborns in the study area.

**Table 2:** Overview of neonatal samples and indicators of population and economy in 2014

Province/city	Hubei	WH	XN	HS	HG	EZ
Pop (10,000)	5815.99	1033.80	248.92	244.92	626.25	105.88
GDP (10 billion CNY)	288.73	100.69	9.64	12.19	14.77	6.87
GDP/Pop (1,000 CNY)	49.64	97.40	38.73	49.77	23.58	64.88
Sample	429,865	68,026	33,036	26,396	52,034	10,289
Sample/Pop (‰)	7.39	6.58	13.27	10.78	8.31	9.72

Province/city	XT	XG	TM	QJ	JZ	JM
Pop (10,000)	116.60	486.13	129.16	95.44	574.42	288.91
GDP (10 billion CNY)	5.52	13.55	4.02	5.40	14.80	13.11
GDP/Pop (1,000 CNY)	47.34	27.87	31.12	56.58	25.77	45.38
Sample	13,180	5,831	12,205	7,606	47,112	19,499
Sample/Pop (‰)	11.30	1.20	9.45	7.97	8.20	6.75

Province/city	XY	SZ	ES	YC	SY	SNJ
Pop (10,000)	560.02	218.38	331.77	410.45	337.27	7.67
GDP (10 billion CNY)	31.29	7.23	6.12	31.32	12.01	0.20
GDP/Pop (1,000 CNY)	55.87	33.11	18.45	76.31	35.61	26.08
Sample	49,306	22,393	32,412	6,047	24,425	68
Sample/Pop (‰)	8.80	10.25	9.77	1.47	7.24	0.89

Pop, total resident population; GDP, gross domestic product; CNY, Chinese yuan

**Fig. 5:**
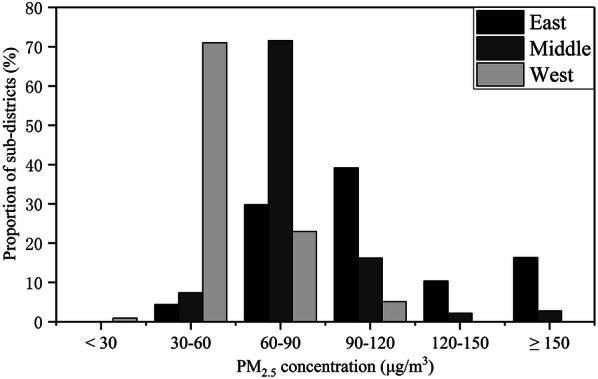
Annual average PM_2.5_ concentration histograms for regions

Table 3 shows the descriptive characteristic of the sample data for the entire province and three regions. The east accounts for greater than half of the newborns, as it is a densely populated area in the province. In contrast, the west has the least newborns. The overall prevalence of PTB is 2.98% in Hubei, and minimal differences are noted among three regions. The majority of pregnant mothers are located in the city proper or general town, but there are distinct differences in the proportion in various urban–rural residence types among the three regions. For instance, the proportion of pregnant women is relatively high in the countryside in the west. Moreover, the PM_2.5_ pollution exposure in the three regions is quite different (Fig. 2). The *q*-statistic in Geodetector (www.geodetector.cn) [65, 66] is 0.38, and the annual PM_2.5_ concentration is significantly different across the regions at the *p *= 0.05 level. Figure 5 shows the average annual PM_2.5_ concentration histograms for the regions, which indicates that the east has wide-ranged and serious air pollution, whereas the middle and west mainly has concentrations of 60–90 μg/m^3^ and 30–60 μg/m^3^, respectively.

**Table 3:** Descriptive characteristics for variables used in multilevel models (%)

Variable	Hubei	East	Middle	West
Total	429,865	228,603	138,310	62,952
GA (weeks)				
Mean (Std)	39.24 (1.27)	39.32 (1.26)	39.14 (1.25)	39.17 (1.32)
< 37	2.98	2.91	2.94	3.32
Residence types				
City proper^a^	31.98	40.04	23.04	22.36
County town	10.45	9.94	9.10	15.24
Suburb	9.10	13.68	4.90	1.70
General town	41.19	31.81	58.65	36.89
Countryside	7.28	4.53	4.31	23.81
PM_2.5_ exposure (30 μg/m^3^)				
Mean (Std)	3.10 (1.18)	3.63 (1.21)	2.87 (0.61)	1.69 (0.53)
Nationality				
*Han* (Main)^a^	96.89	99.63	99.35	81.55
Minority	3.11	0.37	0.65	18.45
Age (years)				
Mean (Std)	26.83 (4.50)	26.95 (4.49)	26.60 (4.32)	26.91 (4.93)
< 20	2.29	1.95	2.08	3.96
20–24	30.49	29.85	32.04	29.39
25–29^a^	43.98	44.22	44.72	41.50
30–34	16.49	17.13	15.38	16.65
≥ 35	6.75	6.85	5.79	8.49
Educational level				
Elementary	19.30	21.21	15.44	20.85
Secondary^a^	74.79	70.70	81.20	75.55
Tertiary	5.91	8.09	3.36	3.59
Frequency of health checks				
< 5^a^	78.31	63.25	95.19	95.91
≥ 5	21.69	36.75	4.81	4.09
Parity				
Firstborn	75.41	73.38	81.01	70.45
Non-Firstborn^a^	24.59	26.62	18.99	29.55
Infant sex				
Male^a^	54.07	54.72	53.82	52.28
Female	45.93	45.28	46.18	47.72
Income (1,000 CNY)				
Mean (Std)	14.32 (7.20)	15.89 (8.15)	13.69 (4.82)	9.98 (5.75)

Std, standard deviation; CNY, Chinese yuan

“^a^” indicates reference group

There is no significant difference in individual-level variables among regions except for nationality and the frequency of physical checks. The west has a greater proportion of minority nationalities than the others, as it includes all ethnic minority autonomous areas in Hubei. Moreover, in the middle and west, the majority of pregnant women have less than five health checks during pregnancy, which might be attributed to the fact that these regions have an under-developed economy and fewer medical facilities. In addition, most people live in general towns and countryside in these regions. Area-level income gaps exist in three regions. The average income in the east is the highest, while that in the west is the lowest.

### Spatial correlation analyses

Table 4 presents the results of the global univariate and bivariate *Moran’s I* in the entire province and three regions. The PM_2.5_ concentration and preterm rate exhibit positive and significant spatial autocorrelation in all cases. The *BiMoran’s I* was close to null in the middle, and a weak positive trend was noted over the entire province and in the west. A strong positive correlation was noted in the east.

**Table 4:** Calculation of results of global Moran’s I (*p* values)

Variable	PM_2.5_ concentration	Preterm rate	Bivariate
Hubei	0.970 (0.00)*	0.861 (0.00)*	0.106 (0.00)*
East	0.968 (0.00)*	0.940 (0.00)*	0.697 (0.00)*
Middle	0.872 (0.00)*	0.856 (0.00)*	− 0.004 (0.44)
West	0.927 (0.00)*	0.731 (0.00)*	0.049 (0.02)*

Significant level: “*” p ≤ 0.05

**Fig. 6:**
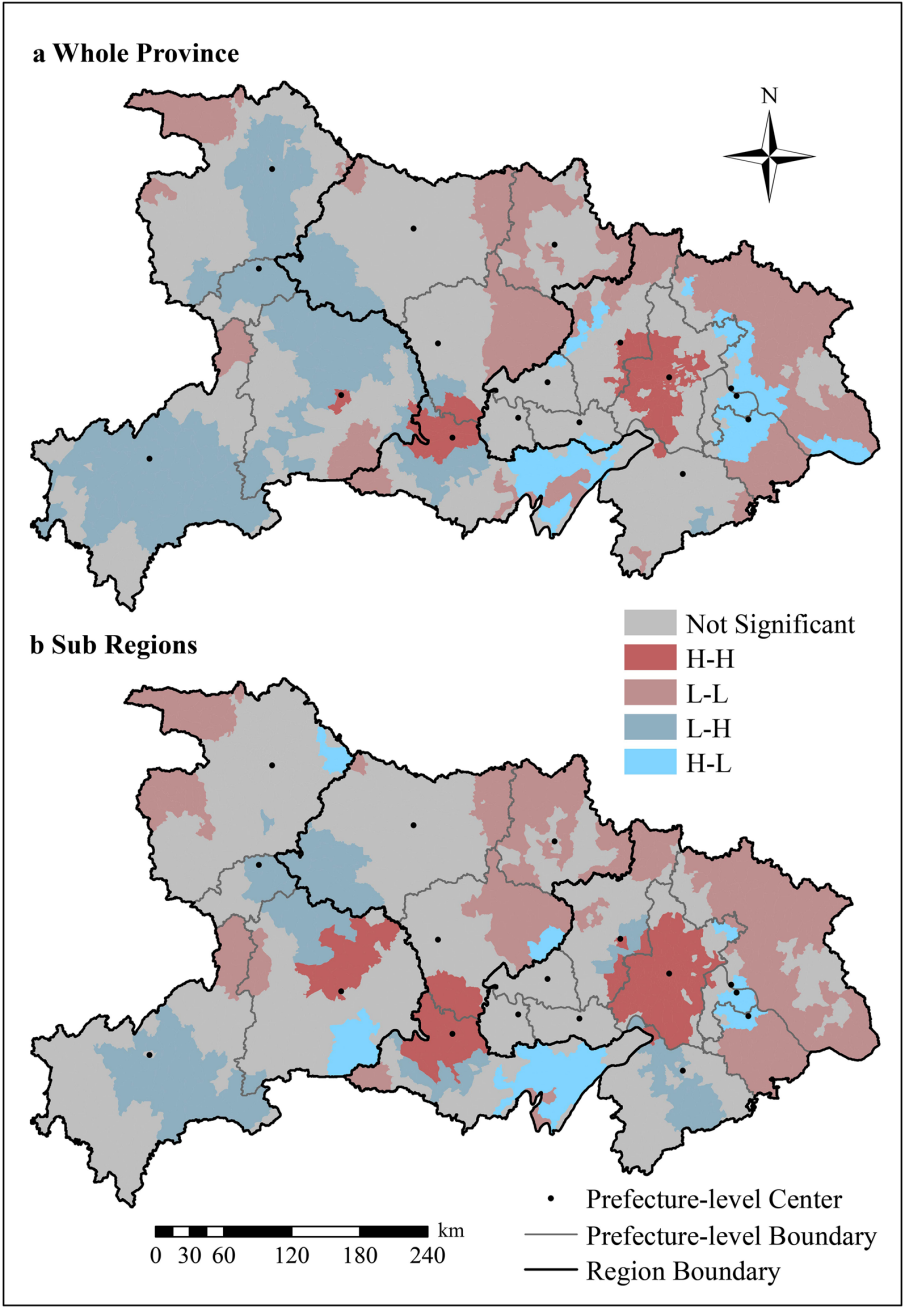
Spatial distribution of bivariate LISA clusters

Figure 6 displays the cluster distribution according to the *BiLISA* value and its significance for the entire province and three regions. “Red” indicates a positive correlation (H–H, L–L) while “blue” indicates a negative correlation (H–L, L–H). From the perspective of the whole province, the H–H areas were concentrated in the east and in JZ in the middle, while L–H areas were mostly located in the west. However, the number of H–L areas was small, and these areas were scattered in the east and middle. However, L–L areas were widely distributed across Hubei. In terms of the regions, four types of areas appeared in each region. H–H and L–L areas dominated the east, while the west had larger L–H areas.

### Multilevel modelling analyses

Table 5 summarizes the results of all of the models related to PM_2.5_ exposure variable. The odds ratio (OR) was calculated as exp (β), and the 95% confidence interval (CI) was reported simultaneously. In the unadjusted full-sample models (model 2), the OR was significantly greater than one (1.06; 95% CI 1.04–1.09) but no longer statistically significant after adjustment by covariates and urban–rural type variables (0.98; 0.95–1.02 in model 4, 0.99; 0.95–1.03 in model 5). The models in the middle exhibited similar trends. All ORs were significant in the eastern-sample models regardless of adjustment. In contrast, the results of all the models in the west were insignificant.

**Table 5:** ORs of PM_2.5_ exposure variable in multilevel models

Region		OR (95% CI)			
		Hubei	East	Middle	West
Model	2	1.06 (1.04–1.09)*	1.13 (1.10–1.16)*	1.15 (1.07–1.23)*	0.93 (0.82–1.07)
	4	0.98 (0.95–1.02)	1.12 (1.06–1.20)*	1.04 (0.96–1.13)	0.88 (0.75–1.04)
	5	0.99 (0.95–1.03)	1.12 (1.05–1.20)*	1.02 (0.94–1.11)	0.83 (0.68–1.00)

OR, odds ratio; CI, confidence interval

Significant level: “*” p ≤ 0.05

**Fig. 7:**
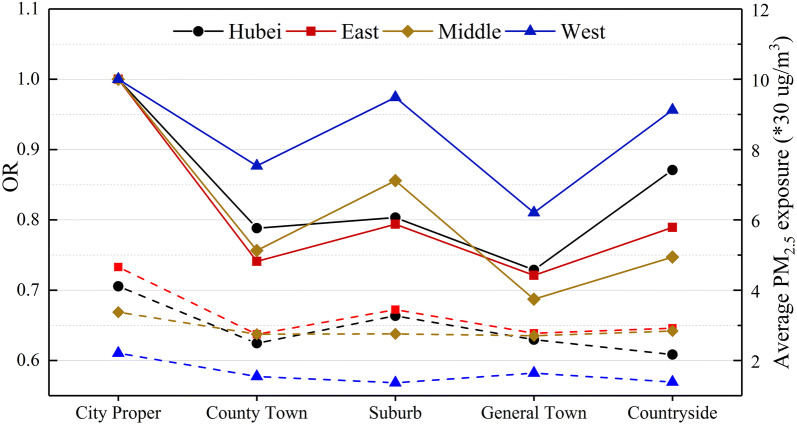
The odds ratios of PTB (solid line) and average PM_2.5_ exposure (dashed line) of five urban–rural types

Table 6 summarizes the results of all multilevel models involving urban–rural type variables. Model 1 was used to test the difference in PTB risk among various residence types. All ORs were less than one, and most were significant. These results indicate that compared with the city proper, other urban–rural types have a lower risk of PTB. However, the effect did not monotonically decrease as urbanity weakened. Figure 7 presents the trends of the PTB risk magnitudes ordered by urbanity from the strongest to the weakest, and W-shapes are noted across the urban–rural continua for the entire province and all regions. After controlling for covariates in model 3 and considering the PM_2.5_ exposure in model 5, the ORs changed slightly compared with model 1. The entire right side of the confidence intervals of ORs approaches or exceeds one, especially in the east and west, which indicates that the differences in PTB risks among urban–rural types have become less obvious.

**Table 6:** ORs of residence type variables in multilevel models

Model	Type	OR (95% CI)			
		Hubei	East	Middle	West
1	County town	0.79 (0.70–0.89)*	0.74 (0.63–0.87)*	0.76 (0.60–0.95)*	0.88 (0.66–1.16)
	Suburb	0.80 (0.73–0.89)*	0.79 (0.71–0.88)*	0.86 (0.68–1.08)	0.97 (0.58–1.62)
	General town	0.73 (0.68–0.78)*	0.72 (0.66–0.78)*	0.69 (0.61–0.78)*	0.81 (0.66–0.99)*
	Countryside	0.87 (0.79–0.96)*	0.79 (0.68–0.92)*	0.75 (0.59–0.94)*	0.96 (0.78–1.18)
3	County town	0.82 (0.72–0.93)*	0.76 (0.64–0.91)*	0.99 (0.77–1.27)	0.74 (0.52–1.07)
	Suburb	0.85 (0.76–0.96)*	0.88 (0.75–1.03)	0.80 (0.64–1.01)	0.99 (0.47–2.10)
	General town	0.78 (0.69–0.87)*	0.82 (0.68–0.99)*	0.74 (0.59–0.94)*	0.80 (0.45–1.44)
	Countryside	0.91 (0.78–1.05)	0.91 (0.73–1.13)	0.77 (0.56–1.06)	0.97 (0.53–1.78)
5	County town	0.81 (0.71–0.92)*	0.82 (0.68–0.99)*	1.01 (0.78–1.30)	0.69 (0.47–1.00)
	Suburb	0.86 (0.76–0.96)*	0.84 (0.71–0.98)*	0.82 (0.64–1.04)	1.15 (0.53–2.48)
	General town	0.78 (0.69–0.88)*	0.79 (0.65–0.96)*	0.75 (0.59–0.94)*	0.96 (0.52–1.79)
	Countryside	0.91 (0.78–1.06)	0.87 (0.70–1.09)	0.77 (0.56–1.06)	1.14 (0.61–2.13)

OR, odds ratio; CI, confidence interval

Significant level: “*” p ≤ 0.05

Table 7 shows the ORs of covariates used in the final model (model 5). No significant difference was observed in the risk of PTB between the *Han* (China’s main nationality) and minority nationalities in Hubei and any region. Pregnant women less than 20 years old have a higher risk of PTB, and the risk continues to increase when pregnant women are over 30 years old. Those with higher education have a significantly higher risk in the west, but no significant differences were found in other cases. Regular health checks can significantly reduce the risk of PTB in the entire province and the east. Firstborn infants in the middle have a higher risk of PTB, but this correlation was not significant in other regions. Female babies have a lower risk than males for the entire province and all regions. No significant non-monotonicity was found for the effect of income on the risk of PTB in any single region, but the direction of the effect varied by region. Specifically, the income could statistically increase the risk of PTB in the full-sample model as well as in the middle. The opposite effect was noted in the west, and the effect was not significant in the east.

**Table 7:** ORs of individual socio-demographic variables in model 5

Variable	OR (95% CI)			
	Hubei	East	Middle	West
Minority (ref: *Han*)	1.04 (0.93–1.16)	0.99 (0.66–1.49)	1.26 (0.88–1.80)	0.95 (0.84–1.08)
Age (ref: 25–29)				
< 20	1.32 (1.18–1.48)*	1.20 (1.01–1.44)*	1.38 (1.13–1.69)*	1.51 (1.21–1.88)*
20–24	0.97 (0.93–1.02)	0.93 (0.87–0.99)*	0.96 (0.89–1.04)	1.18 (1.06–1.33)*
30–34	1.25 (1.19–1.32)*	1.17 (1.09–1.26)*	1.38 (1.26–1.50)*	1.33 (1.17–1.51)*
≥ 35	1.86 (1.75–1.98)*	1.84 (1.69–2.00)*	1.84 (1.64–2.07)*	1.94 (1.68–2.24)*
Education (ref: secondary)				
Elementary	0.97 (0.92–1.02)	0.94 (0.88–1.01)	0.96 (0.87–1.05)	1.08 (0.96–1.22)
Tertiary	0.98 (0.91–1.06)	0.95 (0.87–1.05)	0.95 (0.80–1.14)	1.26 (1.01–1.59)*
Regular health checks (ref: check < 5)	0.89 (0.83–0.95)*	0.84 (0.77–0.91)*	0.91 (0.78–1.08)	1.22 (0.95–1.56)
Firstborn (ref: non–firstborn)	1.04 (0.99–1.09)	0.98 (0.92–1.04)	1.22 (1.12–1.33)*	0.98 (0.89–1.09)
Female (ref: male)	0.79 (0.76–0.82)*	0.78 (0.74–0.82)*	0.79 (0.75–0.85)*	0.84 (0.77–0.92)*
Income	1.03 (0.96–1.10)	0.96 (0.86–1.08)	0.91 (0.77–1.07)	0.94 (0.57–1.52)
Income ^2	1.04 (1.01–1.07)*	1.02 (0.98–1.06)	1.65 (1.34–2.03)*	0.69 (0.52–0.93)*

OR, odds ratio; CI, confidence interval

Significant level: “*” p ≤ 0.05

## Discussion

This study aims to explore the relationships between air pollution and the risks of PTB as well as the risk disparities across different regions and urban–rural continua. We found that the results tend to support the argument that higher pollution exposure during pregnancy can increase the risks of PTB, but some variations were noted in three regions. Spatial analysis showed that the spatial correlation was significantly positive between air pollution and preterm rate in the east and west with an insignificant correlation in the middle. Multilevel models further demonstrated that the risk of PTB increased by 12% (95% CI 7%–18%) for a 30-μg/m^3^ increase in average PM_2.5_ exposure during pregnancy after controlling for covariates and urban–rural variables in the east, while no significant correlation was found in other cases.

Serious air pollution has become a global problem, especially in many developing countries, such as China, India, and countries in western and northern Africa [67]. These conditions significantly threaten human health and living quality, and attention should be given to these issues. Although many studies have investigated the relationships between air pollution and pregnancy outcomes in both developed and developing countries, such as the US [22, 25, 68], Europe [24, 69], China [21, 70, 71] and other countries [72, 73], their findings are inconclusive. Parker et al. hypothesized that these different results may be due to the composition of PM differences and exposure measurement errors [31]. Fleischer et al. suggested that the differences might be related to the threshold effect and the stage of economic development [30]. However, little research has examined the effect of the economy-environment context of location from the perspective of regional and urban–rural disparities in pollution-health relationships.

In fact, another problem is the imbalance in regional and urban–rural development accompanied by the inequality in socioeconomic conditions and differences in environment quality. In the east area in this study, which exhibits the most developed economy and the most serious levels of pollution, the pollution-PTB relationship was significantly positive, but area-level income was not associated with PTB. Conversely, in the west, where the air pollution and economic level were the lowest in the province, increased area-level income could significantly reduce the risk of PTB, while the pollution-PTB relationship was insignificant. Interestingly, the direction of the income effect was positive for PTB in the middle, indicating that economic growth will occur at the expense of health in some cases. We could infer that pollution exposure has a threshold and nonlinear effect [5, 30, 74], and the ranks of importance of income and pollution to health will vary based on geographical context.

Furthermore, the U-shaped pattern of the relationship between economy and environmental quality may explain why the risk of PTB observed in our study does not change monotonically across the urban–rural continuum. We also found that the crude disparity in the risks of PTB followed the same W-shaped pattern across the urban–rural continuum for the entire province and for all regions. In other words, the risks of PTB present a V- or U-shaped structure in urban settings and rural settings. This finding could indicate that an urban disadvantage and rural disadvantage exist simultaneously, and the lowest risk of PTB occurs at a moderate level for both urbanity and rurality. Part of the disparity could be statistically explained by air pollution exposure. In the multilevel models, most of the ORs of other urban–rural types were increased compared with the city proper (though still less than one) when the PM_2.5_ exposure was controlled for. These findings potentially suggest that the air pollution pressure is likely an important aspect of the urban disadvantage. Thus, a finer classification of urban–rural areas is necessary. This information could potentially improve our understanding of the pollution-health relationships and be helpful for governments to develop environmental and public health policy better targeted at appropriate places.

It should be noted that the W-shaped pattern may not always be fixed for other countries and regions, and the shape also depends on the contexts among various urban–rural areas. In China, although some cities have been highly developed (for example, Wuhan is the largest city in central China and one of the mega cities in the whole nation) [75], many rural areas in suburbs and counties are still regarded as poverty-stricken regions with poor living conditions, low-level medical facilities and inconvenient transportation. The economy is driven by energy in most places in China, and thus the economy-environment relationship is mainly located in the left half of the Environmental Kuznets Curve. Similar circumstances likely exist in rapidly developing countries. However, the economy-environment contexts may differ in some developed countries that have experienced suburbanization; thus, some suburbs have more advantages than the city proper in terms of both living conditions and environment quality. Therefore, the urban–rural division should take into account the actual condition of study area. For example, the urban hierarchy and distance to the urban core can be used as important references. In developed countries, several indicators, such as the social deprivation index, may be helpful to finely distinguish different types of suburbs and then further categorize these suburbs into urban–rural types.

Regarding other covariates, this study shows a U-shaped relationship between maternal age and risk of PTB, and firstborn and male babies have a higher risk of PTB than non-firstborn and female infants. Race and ethnicity were often considered in previous studies, but we did not find a significant difference in the risks of PTB between the minority and main nationalities in all regions. Regular examination during pregnancy can reduce the risk of PTB, which is supported by the eastern region results. However, the proportion of pregnant women with regular physical exams in the middle and western regions is very small, which may be related to the incomplete health check records acquired from the hospitals in these regions.

Some limitations should be noted. First, data on some individual-level information, such as occupation, medical history and lifestyle variables, were not considered in the analysis mainly due to the unavailability of these data in the restricted medical record data. Second, given that the income variable is not available in the dataset, we instead used district/county-level annual income, which failed to distinguish the income effect of the five urban–rural types. Fortunately, urban–rural types were treated as dummy variables in the multilevel models, and these data could partly reflect the SES and income effect. Third, other pollutants, such as NO_2_, SO_2_, O_3_ [5, 76] and temperature [77–79], which might also be risk factors of PTB, were not controlled in this study. It should be noted that PM_2.5_ is the main air pollutant in most regions of China, and the spatial variation of temperature in the long term is not notable. As indicated in some prior studies, the adjustment of multi-pollutants and temperature was insignificant [5, 27]. However, without controlling for these factors, the effect of PM_2.5_ might be overestimated. Finally, given the uncertainty and unavailability of where women’s activities took place in daily life, the air pollution concentration of registered residences may not accurately reflect the real air pollution exposure to pregnant women. Future research could measure the mobility-based air pollution exposure for pregnant women and estimate their health effects.

## Conclusions

This study aimed to examine regional and urban–rural disparities in the relationships between PTB risk and air pollution in Hubei Province, China, and to determine how the risk changes as urbanity decreases and to what extent air pollution exposure during pregnancy can explain the variations. Spatial correlation analysis indicated that air pollution exposure and PTB exhibited a significant and positive correlation in areas with serious air pollution burden. Multilevel logistic regressions showed that the risk of PTB in the entire province and all regions followed the same W-shaped pattern as urbanity decreases and rurality increases. The modelling results also found that air pollution exposure during pregnancy could increase the risk of PTB and that a high level of air pollution exposure may be an important disadvantage for urban pregnant women in this setting.

## Data Availability

The datasets collected and analysed in this study are not available in original form due to confidentiality requirements, but part of the anonymized data is available from the corresponding author upon request.

## Abbreviations

PM: Particulate matter
SES: Socioeconomic status
PTB: Preterm birth
LBW: Low birth weight
SGA: Small for gestational age
GA: Gestational age
LMP: Last menstrual period
AQM: Air Quality Monitoring
URCC: Urban-Rural Classification Codes by China National Bureau of Statistics
OR: Odds ratio
CI: Confidence interval
